# B-Type Natriuretic Peptide and Prognosis of End-Stage Renal Disease: A Meta-Analysis

**DOI:** 10.1371/journal.pone.0079302

**Published:** 2013-11-13

**Authors:** Yun-Jiu Cheng, Feng-Juan Yao, Li-Juan Liu, Kai Tang, Xiao-Xiong Lin, Wei-Jie Li, Jing Zhang, Su-Hua Wu

**Affiliations:** 1 Department of Cardiovascular Medicine, The First Affiliated Hospital, Sun Yat-Sen University, Guangzhou, China; 2 Department of Ultrasonography, The First Affiliated Hospital, Sun Yat-Sen University, Guangzhou, China; University of Oxford, United Kingdom

## Abstract

**Background:**

The prognostic importance of B-type natriuretic peptide (BNP) or N-terminal pro BNP (NT-proBNP) in patients with end-stage renal disease (ESRD) remains controversial.

**Methodology/Principal Findings:**

We conducted an unrestricted search from the MEDLINE and EMBASE in all languages that were published between 1966 and Augest2013. Twenty-seven long-term prospective studies met our inclusion criterias. From the pooled analysis, elevated BNP/NT-proBNP was significantly associated with increased all cause mortality [odds ratio (OR), 3.85; 95% CI, 3.11 to 4.75], cardiovascular mortality (OR, 4.05; 95% CI, 2.53 to 6.84), and cardiovascular events (OR, 7.02; 95% CI, 2.21 to 22.33). The funnel plot showed no evidence of publication bias. The corresponding pooled positive and negative likelihood ratio for prediction of all cause mortality were 1.86 (95% CI, 1.66 to 2.08) and 0.48 (95% CI, 0.42 to 0.55), respectively.

**Conclusions/Significance:**

BNP/NT-proBNP is a promising prognostic tool to risk-stratify the patients with ESRD. Further investigations are warranted to elucidate the specific pathogenic mechanisms and the impact of other potential prognostic factors.

## Introduction

Cardiovascular disease is the leading cause of morbidity and mortality in patients with end-stage renal disease (ESRD), accounting for approximately 50% of the deaths [1]. Early identification of ESRD patients at high risk for future events may facilitate more aggressive and focused treatments.

B-type natriuretic peptide (BNP) is a 32-amino acid polypeptide secreted by ventricles of the heart in response to excessive stretching of cardiomyocytes. This peptide is believed to play an important role in regulating blood pressure and volume through direct effects on the kidney and systemic vasculature. BNP is co-secreted along with a 76-amino acid polypeptide, NT-proBNP, which is more stable and biologically inactive [2]. Measurement of circulating natriuretic peptide (NPs), BNP or NT-proBNP has been recommended in the diagnosis and prognosis of patients with acute or chronic heart failure [3]. Given the high prevalence of left ventricular (LV) hypertrophy and systolic dysfunction in patients with ESRD [4], [5], [6], [7], it has been proposed that the NPs may assist in risk stratification for cardiovascular events among patients with ESRD.

The past few years have seen a rapidly growing interest in testing this hypothesis. Many prospective studies have investigated the link between NPs and the adverse outcomes in ESRD patients, and most found a positive association. However, the magnitudes of the association varied between studies and most of them have not been systematically assessed. Several previous relevant reviews involved only about one-fourth of the currently available data [8], [9]. In addition, interpretation of the evidence has been complicated by studies that have involved different markers (ie, BNP, NT-proBNP, or both), different disease outcomes (eg, all-cause mortality, cardiovascular mortality or events) and the impact of other prognostic variables besides NPs. To help resolve this uncertainty of BNP/NT-proBNP as a prognostic tool, our goal, therefore, was to quantify the association between BNP/NT-proBNP and long-term adverse outcomes by conducting a meta-analysis of these prospective studies.

## Methods

The methods used in this review are in accordance with the Meta-Analysis of Observational Studies in Epidemiology: A Proposal for Reporting [10].and follows PRISMA guidelines (Checklist S1).

### Research Objectives

The primary research objective was to determine, by use of systematic review techniques, whether elevated circulating BNP and/or NT-proBNP predicted long-term risks of all-cause mortality, cardiovascular mortality or cardiovascular events (defined as any fatal or nonfatal myocardial infarction, stroke, transient ischemic attack, or heart failure) among patients with ESRD. The secondary objective was to determine the ability of BNP or NT-proBNP to discriminate the patients who do and do not experience subsequent events.

### Data Sources and Selection

To identify relevant studies, MEDLINE (1966- Aug., 2013), EMBASE (1980- Aug., 2013) were reviewed without language restrictions. The electronic searches were completed by manual search of the reports' reference lists. The search strategy, developed with an experienced librarian, included the following terms: 1) end stage, kidney disease, renal disease, dialysis, hemodialysis, peritoneal dialysis, renal replacement therapy; 2) natriuretic peptide, B-type natriuretic peptide, brain natriuretic peptide, N-terminal pro B-type natriuretic peptide, BNP, NT-proBNP); 3) mortality, all-cause mortality, cardiac death, cardiovascular diseases, myocardial ischemia, myocardial infarction, coronary stenosis, cerebrovascular disorders, stroke; and 4) cohort studies, prospective studies, and follow-up studies.

### Study Selection

We first performed an initial screening of titles or abstracts. The second screening was based on full-text review. Studies were considered eligible if they met the following criteria: 1) prospective observational study design; and studies that 2) evaluated the prognosis of patients with abnormal levels of either BNP or NT-proBNP in ESRD patients, 3) examined all cause mortality, cardiovascular mortality or cardiovascular events. We exclude studies that did not report tabular data for the outcomes separately.

### Quality Review

All studies included for methodological and reporting quality were evaluated according to Egger's quality checklist for prognostic studies [11], adapting the checklist for the purposes of this review because all the included studies were prognostic in nature. Two authors (YJ Cheng and FJ Yao) independently rated study quality. Percentage agreement between the 2 reviewers on the items on the quality review ranged from 84% to 100%. Disagreements were resolved by consensus.

### Data Abstraction

Data on the following characteristics were independently extracted with standardized data extraction protocols and corresponded with study authors to obtain supplementary tabular data: study size; geographical location; year of baseline survey; age range of participants at baseline; percentage of female participants; mean duration of follow-up; storage temperature; NPs assay methods; the number and type (all cause death, cardiovascular death and event) of outcome event. In instances of multiple publications, the most up-to-date or comprehensive information was used.

### Data Synthesis and Analysis

Summary estimates of the univariate odds ratio (OR) and likelihood ratios were calculated using the random effects model because fixed and random-effects model results were similar and random-effects models tend to produce more conservative estimates. In the case of papers that gave multiple cut-off points for BNP analysis, the score that gave the maximum overall accuracy was chosen. As studies used different cut-off points for defining raised BNP or NT-proBNP, we calculated Spearman's correlation coefficient between sensitivity and specificity [12]. This looks for the presence of a threshold effect, where variations in sensitivity and specificity are related to differences in the cut-off point used to define an abnormal result. If there is no evidence of a threshold effect then likelihood ratios can be pooled. Consistency of findings across studies was assessed by Cochran's Q and the I^2^ statistic [13]. Heterogeneity was assssed by comparing results from studies grouped according to prespecified study-level characteristics with meta-regression. Small study bias, consistent with publication bias was assessed with funnel plot (i.e. a plot of study results against precision), by Begg's adjusted rank correlation test, and by Egger's regression asymmetry test [14].

All analyses were performed using STATA version 11.0 (Stata Corp LP, College Station, Texas, USA). A P value<0.05 was considered statistically significant.

## Results

### Literature Search

**Figure 1:**
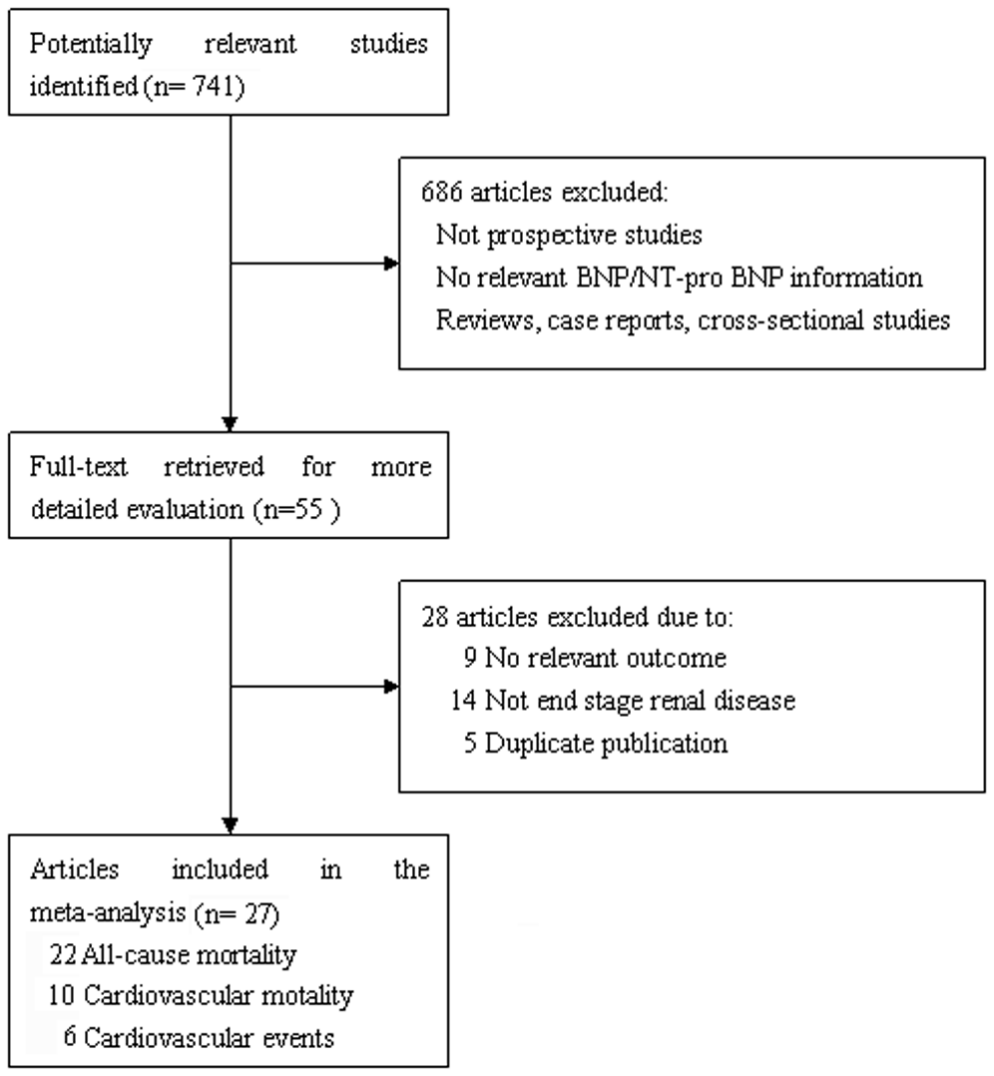
Flow chart of study selection. Flow chart shows literature search for prospective studies of BNP/NT-ProBNP in relation to all-cause mortality, cardiovascular mortality and events in end stage renal disease.

From the search strategy, 741 unique citations were initially retrieved. Of these, the majority were excluded after the first screening based on abstracts or titles, mainly because they were reviews, case-control studies, not BNP/NT-proBNP associated, or not relevant to our analysis. After full-text review of 55 papers, 14 studies were excluded because they recruited non-ESRD patients, and 5 were excluded due to duplicate publications. An additional 9 studies in which the outcomes of interest were not evaluated or not reported separately were also excluded. Finally, 27 studies were included in our meta-analysis (**Figure 1**).

### Methodological Quality

The 27 prospective studies were determined to be of fair quality. Of the primary studies, 59% reported details of exclusion criteria. However, few studies (30%) had explicitly ascertained that patients did not have any acute symptoms of congestive heart failure. The majority of studies (96%) reported at least 1 cardiac risk factor, and 77% reported baseline cardiovascular history. However, only 37% of studies indicated the participants' baseline medication use. All the studies described the BNP/NT-proBNP assays and reported cutoff values to define normal and abnormal levels. BNP/NT-proBNP values were known for all patients. All the studies described treatments or management of patients during follow-up. Only a minority (30%) of primary studies specified the number of patients lost to follow-up, with 22% reporting that no patients were lost to follow-up.

### Study Characteristics

Twenty-seven relevant studies reporting on 8,666 individuals were identified from 16 countries. Thirteen studies were based in Europe [4], [7], [15], [16], [17], [18], [19], [20], [21], [22], [23], [24], [25], 5 in North America [5], [26], [27], [28], [29], [30], 2 in Australia [31], [32] and and 6 in East Asia [6], [33], [34], [35], [36], [37]. From all primary studies, over 63.4% of patients were receiving hemodialysis (HD). Of the total population, 57.1% were males and the median (or mean) ages of the cohorts ranged from 48.6 to 70 years. The mean duration on dialysis was 36 months and the patients followed for an average of 23 months (range, 12 to 60 months). Fifteen studies reported associations with NT-proBNP only, 8 with BNP only, and 4 with both NPs. The median value of NT-proBNP and BNP levels varied substantially across available studies (NT-proBNP values ranged from 288 to 9761 pg/mL; BNP values ranged from 116.8 to 570 pg/mL), with a tendency toward higher values in individuals with higher incidence of cardiovascular disease at entry. **Table 1** **and** **Table 2** demonstrates variability in prevalence of diabetes mellitus (or diabetic nephropathy), prior history of cardiovascular disease, length of follow-up of individual studies and mean number of years spent on dialysis, characteristics of the assays and outcomes across the individual studies.

**Table 1:** Summary of Available Prospective Studies Included in the Present Meta-analysis.

Source Study	Location	No. n	Mean Age, y	Male, %	patient type	Mean time On HD/PD†, mo	Prior History of CAD or MI*	Diabetes Mellitus	Duration of Follow-up	Lost to Follow-up
							%	%	Mo	%
Kim YK [33]	Korea	72	49	52.8	HD	38	…	15	45	0
Koch M [15]	Gernany	255	69.6	60	HD/PD	…	52.2	45	41	0
Roberts MA [31]	Australia	108	62.3	64	HD/PD	30	31	20	33.6	0
Zoccali C [7]	Italy	246	60.2	53.9	HD/PD	43	47.56	15	37	0
Codognotto M [16]	Italy	50	68	72	HD	…	…	…	36	0
Goto T [34]	Japan	53	61	56.6	HD	…	…	34	12	0
Hallen J [17]	Norway	107	62	75	HD	20	51	35	48	0
Apple FS [26]	USA	399	61	58	HD	24	30	46	24	0
Paniagua R [27]	Mexico	753	48.6	55.1	HD/PD	…	…	…	16	0
Svensson M [18]	Denmark	206	67	27	HD/PD	30	68.5	22	24	0
Trape J [19]	Spain	52	74	46	HD	42.5	48	28	36	0
Wang AY [6]	Hongkong	230	56	51	PD	…	22.6	30	36	0
Gutierrez OM [28]	USA	2990	63.2	55.2	HD	…	21.1	50	12	0
Guo Q [20]	Sweden	222	63.8	55.7	HD	27.9	64	26	31	0
Madsen L [4]	Denmark	109	61.8	75.2	HD	20	50.5	34.9	32	0
Hickman PE [32]	Australia	143	59.7	63	HD/PD	40.6	37.8	26.6	30	0
Sun L [35]	China	217	65.4	62.2	HD	…	56.7	47.5	24	0
Paniagua R [29]	Mexico	965	47.3	58.3	PD	…	…	43.32	32	9.3
Satyan S [5]	USA	150	56	52	HD	…	47	31	34	0
Sharma R [21]	UK	140	52	64.3	HD/PD	…	29	38	39	0
Ishii J [36]	Japan	100	58	61	HD/PD	48	22	41	24	0
Biasioli S [22]	Italy	52	58.7	73.1	HD	60	62.5	19.2	28	0
Naganuma T [37]	Japan	164	58.8	70.1	HD	…	77.4	36.6	36	0
Sivalingam M [23]	UK	103	50.2	68	HD	57.5	48.5	…	48	4.85
Selim G [24]	Macedonia	125	53	57.6	HD	75.2	…	13.6	24	0
Westenbrink BD [25]	Netherlands	59	70	52.5	HD	25	25.4	30.5	35	0
Foley RN [30]	Canada	**596**	**51.5**	60.4	HD	9	…	17.8	24	0

† HD indicates hemodialysis, PD, peritoneal dialysis.

* CAD indicates coronary artery disease, MI indicates myocardial infarction

**Table 2:** Characteristics of the Assays and Outcomes.

Source Study	Peptides	Median	Assay	All-cause	Cardiovascular	Cardiovascular
	Assessed	Peptide, pg/ml	Source	Mortality n/100 patient-years	Mortality n/100 patient-years	Events n/100 patient-years
Kim YK [33]	NT-proBNP	6165	Roche	2.96	0.37	4.07
Koch M [15]	BNP	340	Biosite	9.64	1.38	8.15
Roberts MA [31]	BNP/NT-proBNP	191/3233	Biosite/Roche	6.29	0.99	7.95
Zoccali C [7]	BNP	361	Peninsula	8.30	4.61	9.75
Codognotto M [16]	NT-proBNP	9719	Dade Behring	8.67	…	…
Goto T [34]	BNP	390	Shionogi	…	11.3	24.5
Hallen J [17]	NT-proBNP	3912	Roche	12.15	…	…
Apple FS [26]	NT-proBNP	4032	Roche	12.66	…	…
Paniagua R [27]	NT-proBNP	5700¶	Roche	18.13	8.47	…
Svensson M [18]	NT-proBNP	12200‡	Roche	21.84	…	…
Trape J [19]	NT-proBNP	33314‡	Roche	17.95	6.41	…
Wang AY [6]	NT-proBNP	5698	Roche	9.56	6.23	11.30
Gutierrez OM [28]	NT-proBNP	5100	Roche	14.78	8.29	…
Guo Q [20]	NT-proBNP	9761	Siemens	14.81	5.92	24.74
Madsen L [4]	NT-proBNP	4079	Roche	11.68	3.09	…
Hickman PE [32]	BNP/NT-proBNP	116.8/591	In house/Roche	7.82	…	…
Sun L [35]	BNP/NT-proBNP	570/725	Roche/in house	…	5.99	28.11
Paniagua R [29]	NT-proBNP	6198	Roche	12.27	4.24	…
Satyan S [5]	NT-proBNP	3276	Roche	10.82	6.12	…
Sharma R [21]	NT-proBNP	350‡	Roche	4.62	3.08	…
Ishii J [36]	BNP	200	Shionogi	9.50	6.00	…
Biasioli S [22]	BNP	335	Meia	7.44		
Naganuma T [37]	BNP	450	Shionogi	…	2.64	…
Sivalingam M [23]	BNP/NT-proBNP	447/677‡	Biomed/Roche	8.49	…	…
Selim G [24]	BNP	1200‡	In house	11.20	7.60	…
Westenbrink BD [25]	BNP	303	Biosite	14.53	…	…
Foley RN [30]	NT-proBNP	288	Roche	2.77	1.76	6.54

‡ Best cut-off;

¶ Mean

### Association with All-cause Mortality

**Figure 2:**
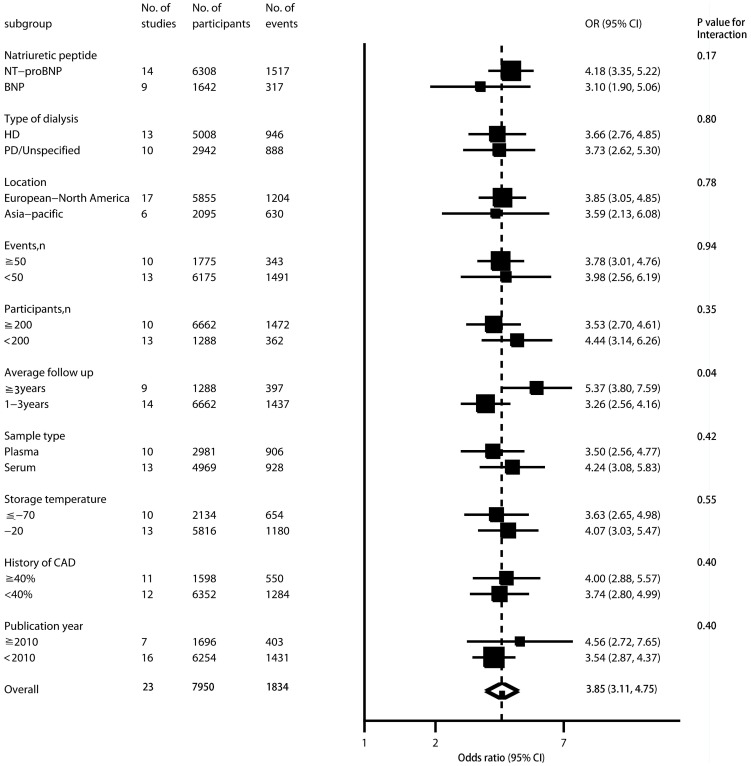
Association between elevated BNP and all cause mortality in patients with end stage renal disease, according to different study level characteristics.

There were 22 primary studies evaluating the association between BNP and all-cause mortality. In the forest plot of individual prognostic effect sizes in Table 3, Figure 2, Figure S1 and Table S1 the lower boundaries of the 95% CIs of almost trials were greater than 1, suggesting a consistent association between BNP and all cause mortality. From the pooled analysis, elevated BNP was significantly associated with increased all-cause mortality (OR, 3.57; 95% CI, 3.17 to 4. 02). There was statistical heterogeneity between studies (I^2^ = 55.81%, 95% CI, 29.45% to 72.33%, P = 0.001). However, little of the heterogeneity was explained by type of NP assayed, type of dialysis, location, number of participants and events, sample type and storage temperature, percentage of coronary artery disease at baseline, publication year. Studies that followed up more than 3 years tended to report somewhat higher ORs than studies with shorter follow-up duration (P = 0.04; Figure 2).

**Table 3:** Summary estimates of odds ratios and likelihood ratios to predict all cause mortality, cardiovascular mortality or cardiovascular events.

Summary estimates	No. of studies	No. of participants	No. of events	OR (95% CI)	Sensitivity (95% CI)	Specitivity (95% CI)	PLR (95% CI)	NLR (95% CI)
All cause mortality	23	7,950	1,834	3.85 (3.11, 4.75)	0.70 (0.65, 0.74)	0.63 (0.58, 0.67)	1.86 (1.66, 2.08)	0.48 (0.42, 0.55)
Cardiovascular mortality	10	6,396	689	4.05 (2.53, 6.84)	0.75 (0.67, 0.81)	0.60 (0.53, 0.66)	1.87 (1.52, 2.30)	0.42 (0.30, 0.58)
Cardiovascular events	6	1,463	425	7.02 (2.21, 22.33)	0.80 (0.65, 0.89)	0.64 (0.49, 0.76)	2.21 (1.48, 3.30)	0.32 (0.17, 0.58)

CI, confidence interval; OR, odds ratio; PLR, positive likelihood ratio; NLR, negative likelihood ratio.

**Figure 3:**
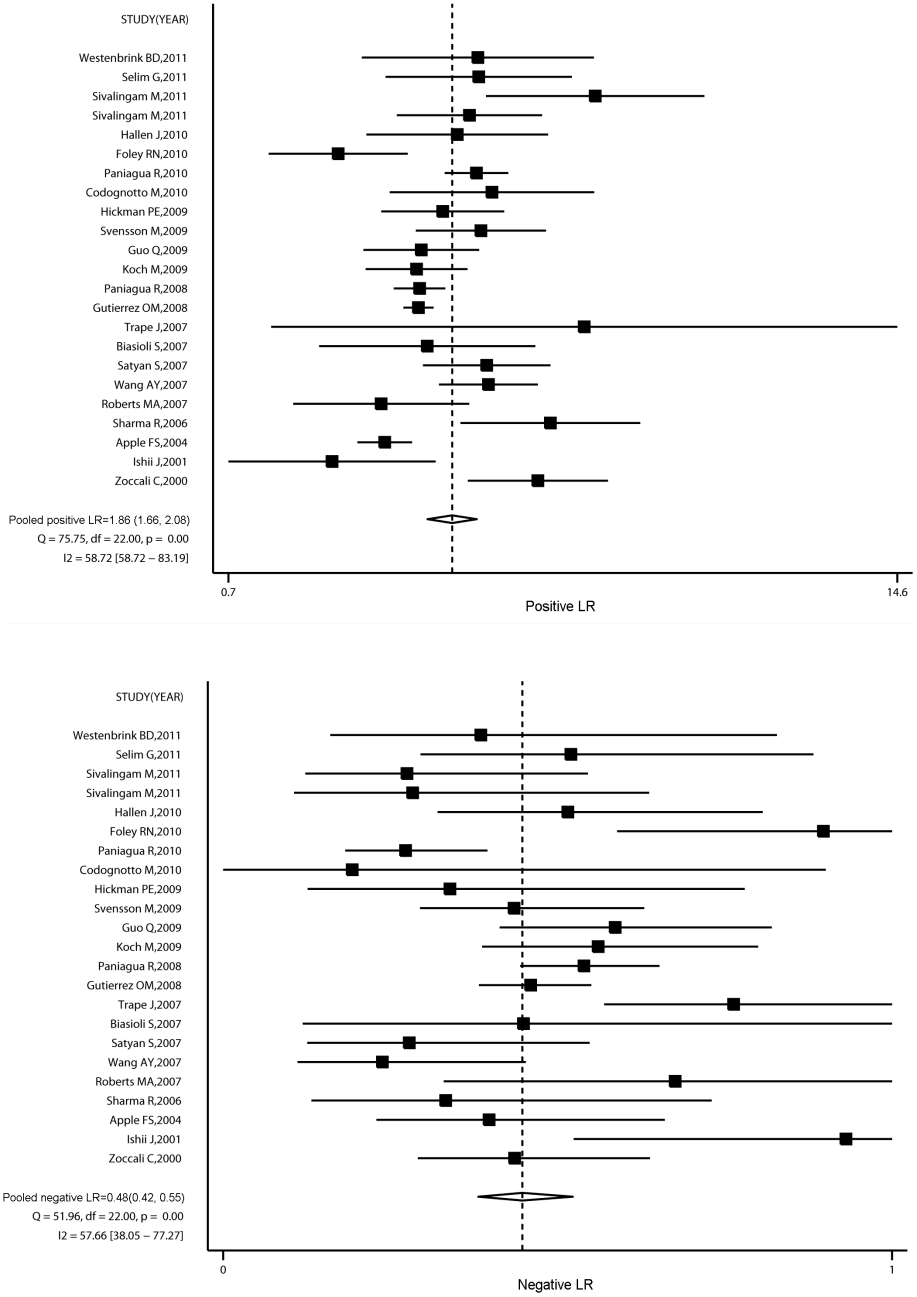
Summary of likelihood ratios of an elevated BNP to predict all cause mortality.

The sensitivity and specitivity of increased BNP or NT-proBNP levels to predict all cause mortality were 0.70 (95% CI, 0.65 to 0.74) and 0.63 (95% CI, 0.58 to 0.67), respectively. Spearman's correlation between sensitivity and specificity was 0.08 (P = 0.70), suggesting no evidence of a threshold effect. We therefore calculated pooled positive (PLR) and negative likelihood ratios (NLR). The summary PLR was 1.86 (95% CI, 1.66 to 2.08) and NLR was 0.48 (95% CI, 0.42 to 0.55). There was statistical heterogeneity for the PLR (I^2^ = 58.72%, 95% CI, 58.72% to 83.19%, P = 0.001) and NLR (I^2^ = 57.66%, 95% CI, 38.05% to 77.27%, P = 0.001) (Figure 3).

**Figure 4:**
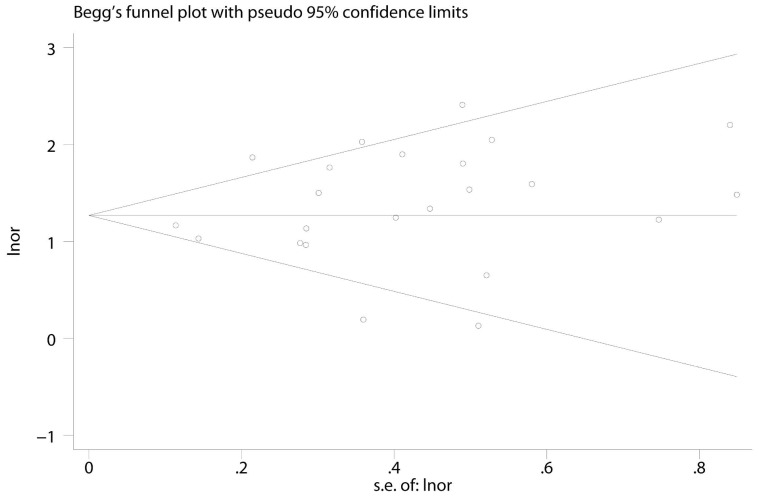
Begg's funnel plots with 95% CI for BNP or NT-proBNP primary studies.

Visual inspection of the Begg funnel plot did not identify substantial asymmetry. The Begg rank correlation test and Egger linear regression test also indicated no evidence of publication bias among studies of either NP and all-cause mortality (Begg, P = 0.49; Egger, P = 0.22) (Figure 4).

### Association with Cardiovascular Mortality or Events

The elevated BNP/NT-proBNP was associated with increased cardiovascular mortality (OR: 4.05; 95% CI, 2.53 to 6.84) with high heterogeneity (I^2^ = 80.38%, 95% CI, 64.77% to 89.07%, P<0.001) (Table 3, Figure S2 and Table S1). When we removed the study by Foley and colleagues that reported a lower OR in a large population [30], the OR for the remaining studies did not materially change (4.58; 95% CI: 3.40 to 6.17; P<0.001), but heterogeneity was decreased to 36.56% (95% CI: 0.00% to 70.81%; P = 0.13).

There were 6 studies that reported the association with cardiovascular events. Elevated BNP/NT-proBNP was strongly associated with a significant increase in long-term cardiac events (OR:7.02; 95% CI: 2.21 to 22.33; P<0.001) (Table 3, Figure S3 and Table S1). BNP/NT-proBNP had better pooled sensitivity (0.80 vs 0.75), specificity (0.64 vs 0.60), PLR (2.21 vs 1.87) and NLR (0.32 vs 0.42) for cardiovascular events prediction than for cardiovascular mortality prediction (**figure S4, figure S5**).

## Discussion

The present meta-analysis of more than 8000 patients with ESRD from 27 long-term prospective studies provides evidence that elevated BNP/NT-proBNP predicted approximately a four-fold increase in the risk of all cause mortality and cardiovascular mortality, and over a seven-fold increase in the risk of cardiovascular events.

Although NT-proBNP is more stable with longer half-life than BNP (≈120 minutes versus ≈20 minutes, respectively) [2] and has theoretical superiority for prognostication, the present data suggest that a given proportional increment in each marker is similarly associated with increased risk of all-cause mortality. On the basis of indirect comparisons with previous meta-analysis of several established risk factors, the magnitude of death risk with BNP/NT-proBNP concentration appears to be stronger than those with C-reactive protein, albumin, troponin or homocysteine [38], [39], [40].

Understanding the mechanisms that underlie elevation in BNP/NT-proBNP levels among ESRD patients is of particular importance to help frame appropriate therapeutic decisions. Previous studies have suggested that the mechanism for a rise in NPs among ESRD patients was a result of LV structural and functional abnormalities [6], [41], enabling NPs to be a potential marker of LV hypertrophy in ESRD [42], [43]. In addition,there is emerging evidence that increases in circulating NPs may indicate myocardial ischemia or necrosis, due to the coronary atherosclerosis prevalent in patients with ESRD [6], [44], supported by a strong relationship between elevated BNP/NT-proBNP and cardiovascular mortality/events in our analysis. Furthermore, given that BNP and NT-pro-BNP are released in response to increased myocardial wall stress, it is tempting to hypothesize that circulating NPs levels may be a useful marker of volume status in ESRD patients [45], [46]. The fluid overload may affect the intensity of chronic inflammation through the presence of intestinal wall edema, which allows a translaocation of bacteria endotoxin from intestinal lumen to bloodstream and contribute to an increased mortality in patients with ESRD [47]. However, other studies failed to detect a positive association between fluid removed and change of BNP in patients receiving HD [41], [48], suggesting that this mechanism needs to be further elucidated.

There is one factor that may confound the interpretation of elevated NPs in ESRD patients. Previous authors have suggested that BNP and NT-proBNP concentrations are strongly correlated with residual renal function [49], [50], making both NPs potentially lose prognostic value in ESRD patients. Nevertheless, several latest studies have reported both NPs are only 20% or less dependent on renal function for their clearance [51], [52], [53], indicating BNP/NT-proBNP to be a prognostic marker for patients with ESRD.

Along with the strict inclusion criteria, strengths of this meta-analysis include the large number of patients analyzed, the robustness of the findings at sensitivity analyses, the fact that all subgroup analyses were prespecified a priori. The absence of important publication bias supports the robustness of the study findings. Although the present study involves over 4 times as much information as in previous reviews [8], [9], it has been limited by the moderate amount of available data from primary studies. For example, of the 27 studies, risk estimates for cardiovascular mortality and events were only available from 10 and 6 studies, respectively. The studies included in our analysis used different cut-off points to define high-risk, which varied significantly between studies. In the analysis with all cause mortality as the end-point, the ‘best-fit’ cut-off point ranged from surprisingly low to expectedly high in different studies. This variation in cut-off point is at least in part related to the significant heterogeneity in patient population between the different studies. From the currently available data it is not possible to draw any firm conclusions regarding the most appropriate cut-off point for risk stratification. Another acknowledged limitation to the meta-analysis is the inability to adjust statistically for identical covariates or prognostic factors that could have influenced the outcomes of ESRD. In addition, although serum concentration of BNP is associated with residual renal function and left ventricular dysfunction, the limited data of glomerular filtration rate and left ventricular ejection fraction reported in the original studies made it difficult to conduct a further statistical investigation.

BNP/NT-proBNP is a promising prognostic tool to risk-stratify stable, asymptomatic ESRD patients, as elevated levels identify a subset of ESRD patients who have poor survival and higher risk of cardiovascular events. This study corroborates previous postulates that a cardiovascular cause underlies the association between mortality and elevated BNP or NT-proBNP in ESRD patients. Further studies, including well-designed clinical trials, are warranted to elucidate the specific pathogenic mechanisms, and the impact of other potential prognostic factors.

## Supporting Information

Figure S1 **Association between elevated BNP and all cause mortality in patients with end stage renal disease.**
(TIF)

Figure S2 **Association between elevated BNP and cardiovascular mortality in patients with end stage renal disease.**
(TIF)

Figure S3 **Association between elevated BNP and cardiovascular events in patients with end stage renal disease.**
(TIF)

Figure S4 **Summary of likelihood ratios of an elevated BNP to predict cardiovascular mortality.**
(TIF)

Figure S5 **Summary of likelihood ratios of an elevated BNP to predict cardiovascular events.**
(TIF)

Table S1 **Data set of tabular data and odds ratio calculations for all cause mortality, cardiovascular mortality and cardiovascular events.**
(XLS)

Checklist S1 **PRISMA Checklist.**
(DOC)
