# Supplementary figures and images for: B-Type Natriuretic Peptide and Prognosis of End-Stage Renal Disease: A Meta-Analysis
Source: PLoS One. 2013 Nov 13;8(11):e79302. doi: 10.1371/journal.pone.0079302 (PMC3827377; doi:10.1371/journal.pone.0079302)

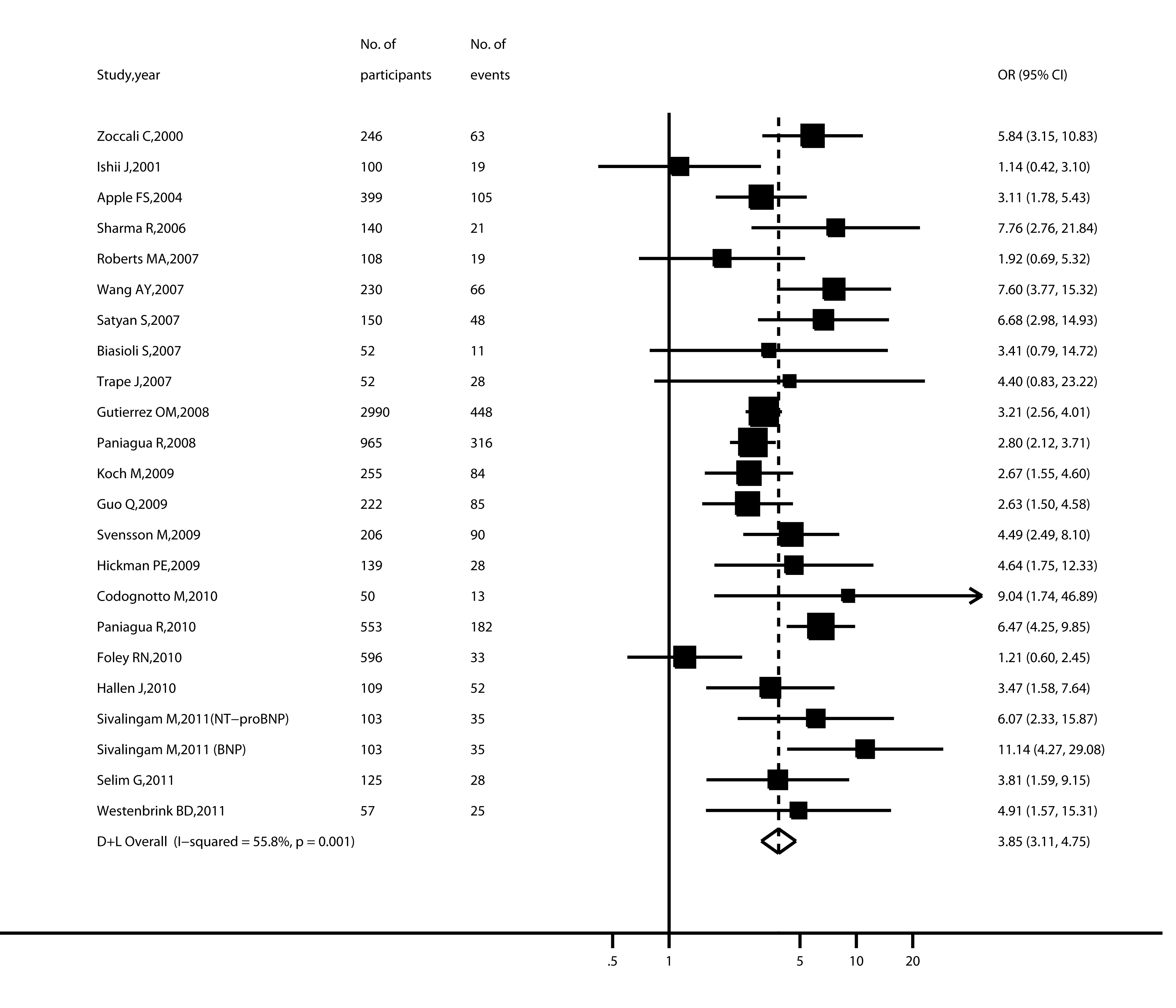

Supplement: Figure S1 — Association between elevated BNP and all cause mortality in patients with end stage renal disease. (TIF) [file pone.0079302.s001.tif]

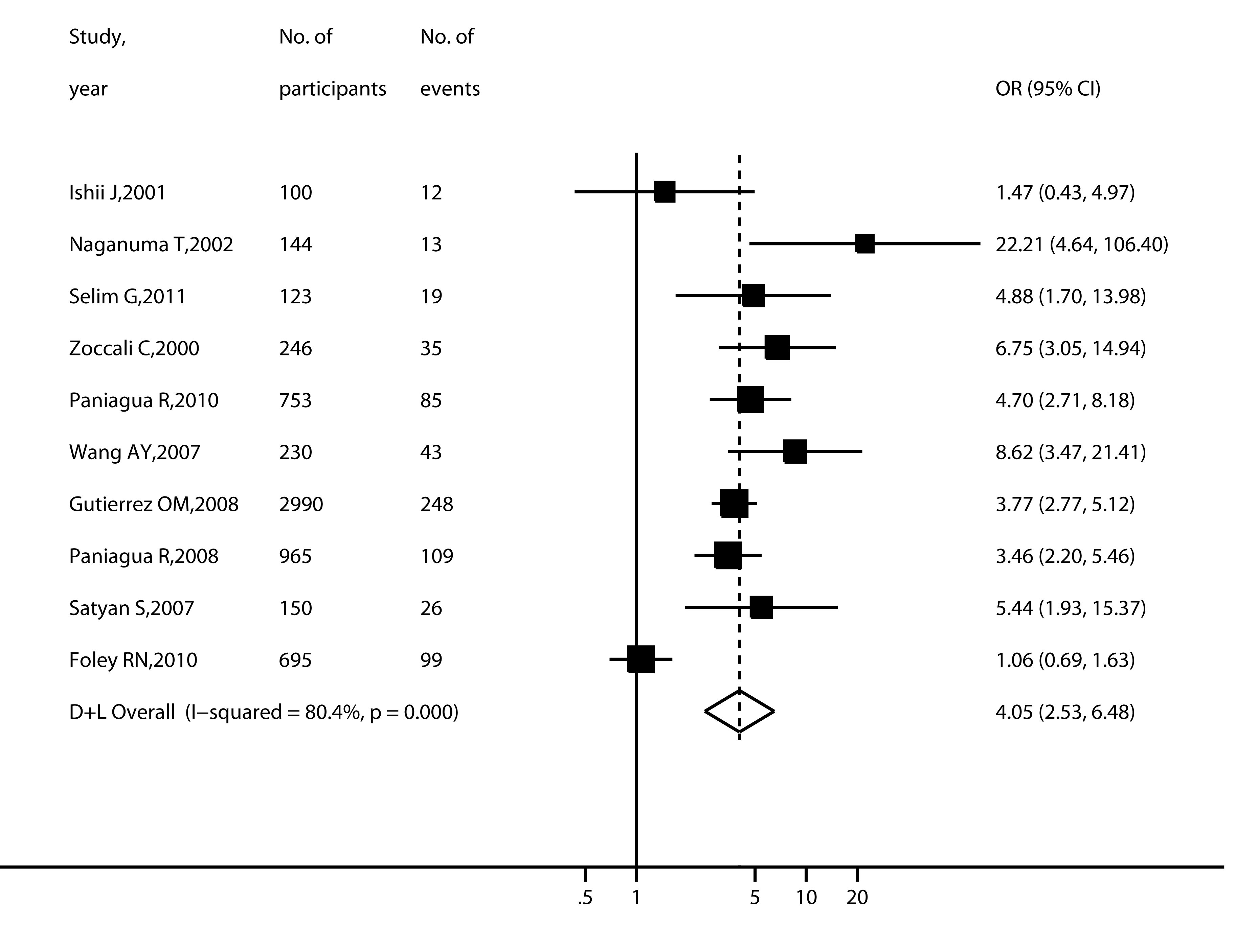

Supplement: Figure S2 — Association between elevated BNP and cardiovascular mortality in patients with end stage renal disease. (TIF) [file pone.0079302.s002.tif]

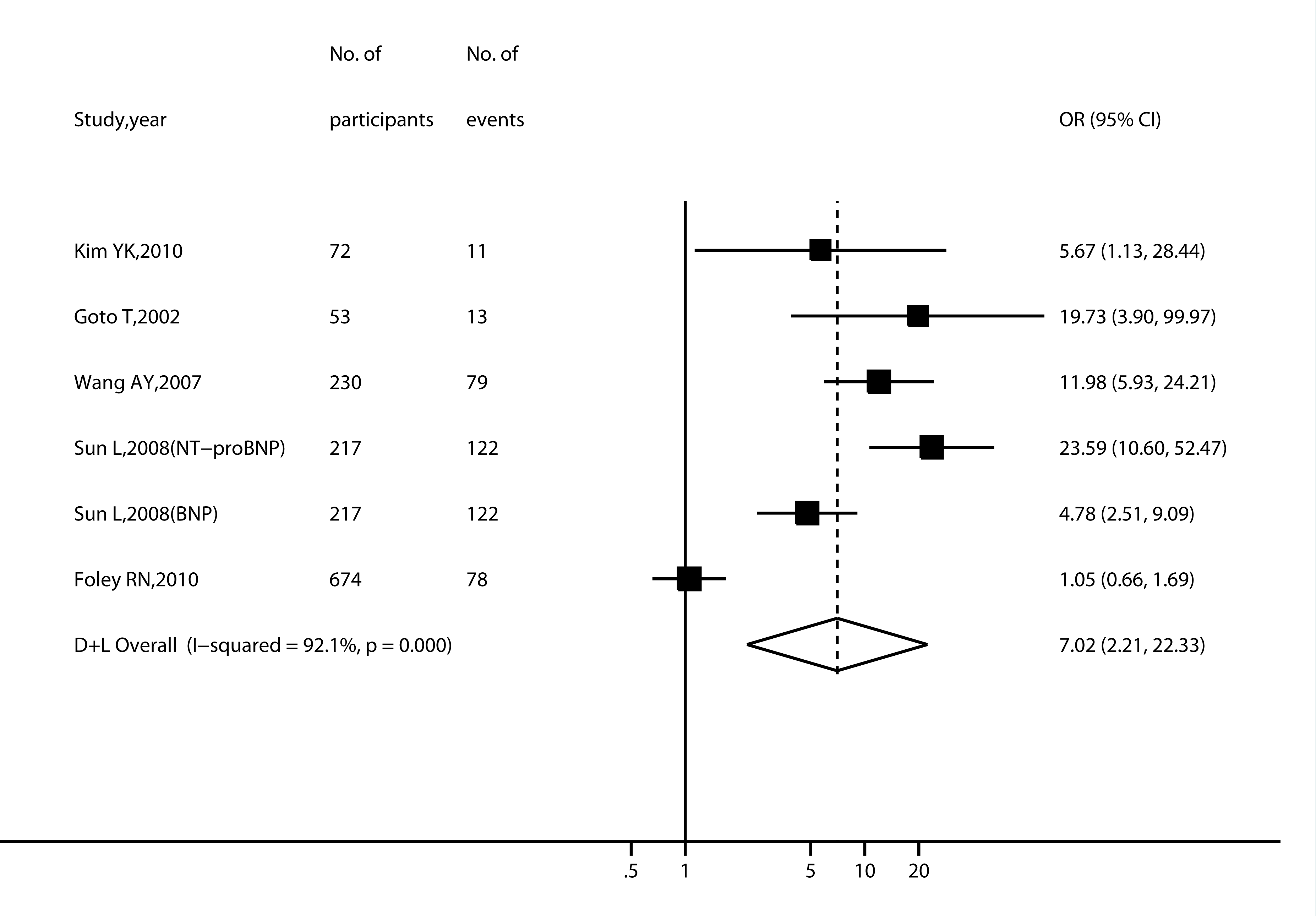

Supplement: Figure S3 — Association between elevated BNP and cardiovascular events in patients with end stage renal disease. (TIF) [file pone.0079302.s003.tif]

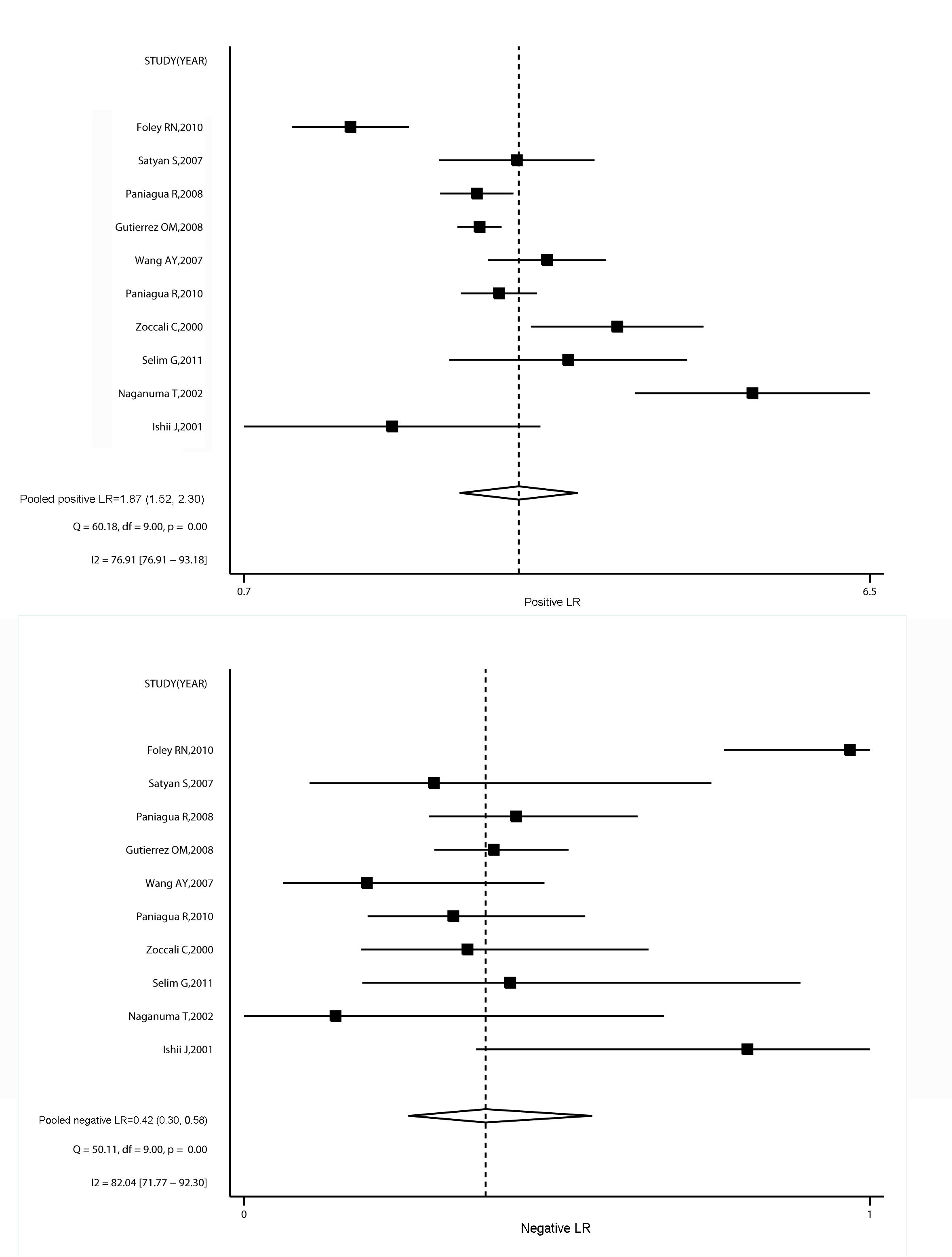

Supplement: Figure S4 — Summary of likelihood ratios of an elevated BNP to predict cardiovascular mortality. (TIF) [file pone.0079302.s004.tif]

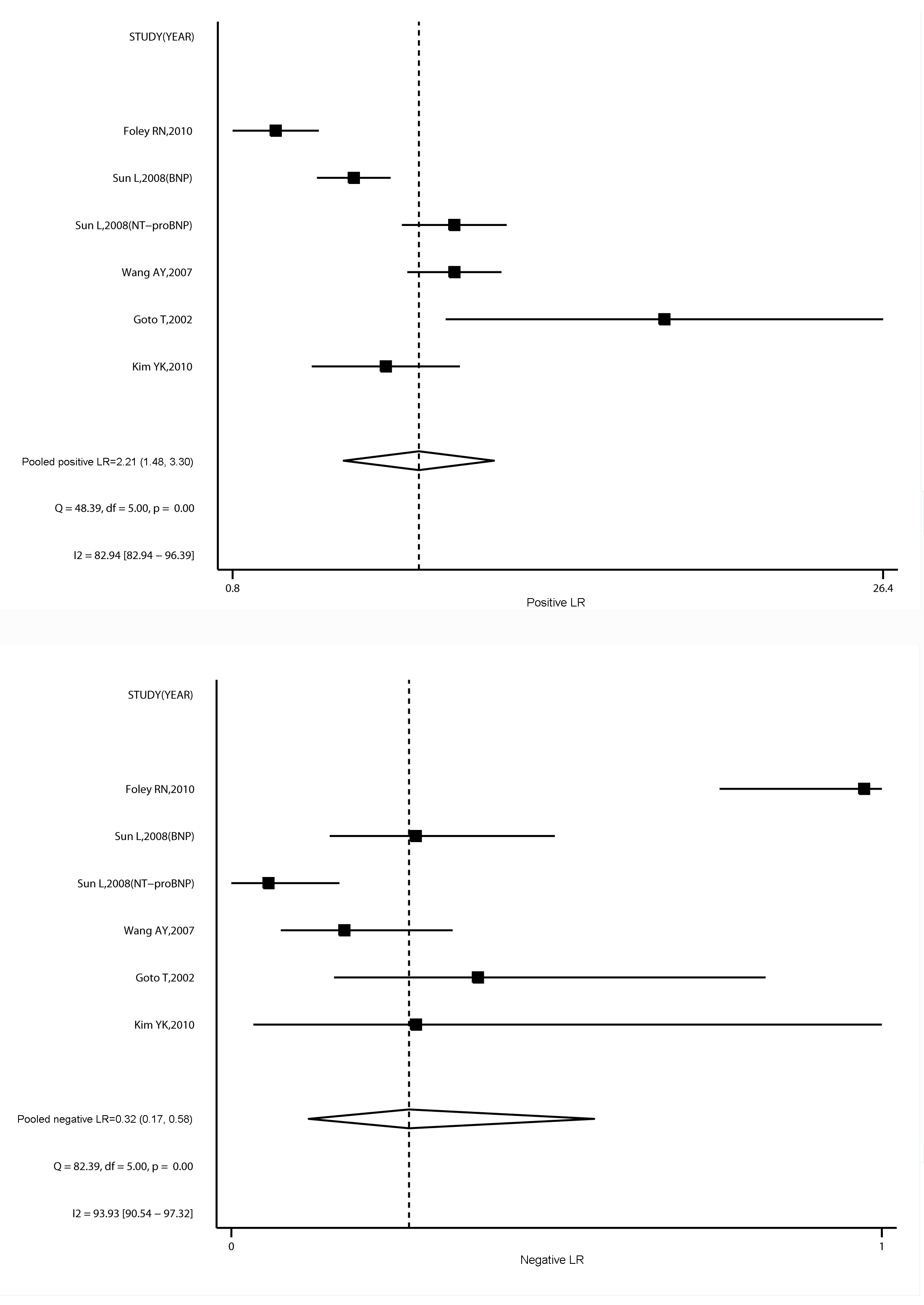

Supplement: Figure S5 — Summary of likelihood ratios of an elevated BNP to predict cardiovascular events. (TIF) [file pone.0079302.s005.tif]
